# Serum IGFBP-2 and Risk of Atypical Hyperplasia of the Breast

**DOI:** 10.1155/2015/203284

**Published:** 2015-05-28

**Authors:** Chelsea Catsburg, Marc J. Gunter, Lesley Tinker, Rowan T. Chlebowski, Michael Pollak, Howard D. Strickler, Michele L. Cote, David L. Page, Thomas E. Rohan

**Affiliations:** ^1^Department of Epidemiology and Population Health, Albert Einstein College of Medicine, Bronx, NY 10461, USA; ^2^Department of Epidemiology and Biostatistics, School of Public Health, Imperial College, London W2 1PG, UK; ^3^Division of Public Health Sciences, Fred Hutchinson Cancer Research Center, Seattle, WA 98109, USA; ^4^David Geffen School of Medicine, University of California Los Angeles, Los Angeles, CA 90095, USA; ^5^Department of Oncology, McGill University, Montreal, QC, Canada H3A 0G4; ^6^Population Studies and Prevention Program, Karmanos Cancer Institute, Wayne State University, Detroit, MI 48201, USA; ^7^Department of Pathology, Vanderbilt University Medical Center, Nashville, TN 37232, USA

## Abstract

Atypical hyperplasia of the breast (AH) is associated with increased risk of subsequent invasive breast cancer, yet little is known about the etiology of AH. Insulin-like growth factor binding protein 2 (IGFBP-2) may contribute to the development of AH due to its proliferative effects on mammary tissue. We conducted a nested case-control study of postmenopausal women enrolled in Women's Health Initiative-Clinical Trial. Cases were 275 women who developed incident AH during follow-up, individually (1 : 1) matched to controls. Levels of IGFBP-2 were determined from fasting serum collected at baseline. Multivariable conditional logistic regression models were used to estimate odds ratios for the association of IGFBP-2 with risk of AH. Serum IGFBP-2 was associated with a nonsignificant decrease in risk for AH, when comparing the highest quartile to lowest quartile (OR = 0.65; 95% CI = 0.32–1.31). This decrease in risk was most evident when analyses were restricted to nondiabetic, nonusers of hormone therapy (OR = 0.33, 95% CI = 0.13–0.86, *p*_trend_ = 0.06) and nondiabetic women who were overweight or obese (OR = 0.43, 95% CI = 0.18–1.03, *p*_trend_ = 0.05). Results from this study provide some support for an inverse association between serum IGFBP2 levels and risk of AH, particularly in nondiabetic women who are overweight or obese. Further studies are required to confirm these results.

## 1. Introduction

Women with benign proliferative breast disease (BPBD) have an approximately twofold overall increase in breast cancer risk which is particularly high (fivefold increase) in women who have proliferative disease with atypia (i.e., atypical hyperplasia (AH)) [1–4]. This finding is in line with the multistep model of breast cancer development, which posits that invasive carcinoma arises via a series of steps in which nonatypical proliferative changes and proliferative disease with AH precede the development of carcinoma* in situ* and ultimately invasive cancer [5, 6].

Little is known regarding the etiology of AH and the progression to breast cancer. However, insulin-like growth factors and their binding proteins have been shown to be critical in the development of many epithelial cancers, including breast cancer [7]. Insulin-like growth factor binding protein 2 (IGFBP-2) is of particular interest as* in vitro* studies have shown that it has a proliferative effect in mammary and other tumor cell lines [8–10] and clinical studies have shown it to be a prognostic biomarker for many cancers [11]. Thus, serum level of IGFBP-2 is a plausible marker for the progression from benign proliferative breast disease to proliferative disease with AH and ultimately to breast cancer [9].

Potentially counterbalancing the proliferative effect of IGFBP-2 is the observation that serum IGFBP-2 levels are inversely associated with obesity [12]. Obesity is a well-established risk factor for postmenopausal breast cancer and may also be a risk factor for BPBD and AH [13]. The association of obesity with risk is thought to be mediated by elevated circulating estradiol levels [14–16]; IGFBP-2 has been shown to be inversely associated with serum estradiol [17]. IGFBP-2 levels have also been inversely correlated with weight, development of adipose tissue, fasting insulin levels, and development of type II diabetes [12, 18, 19]. Previously, we found serum levels of other adiposity-related markers, specifically relatively high levels of insulin, estradiol, and C-reactive protein and relatively low levels of adiponectin, to be associated with increased risk of BPBD including AH [14].

These observations suggest a potential dual effect for IGFBP-2 in breast cancer development: IGFBP-2 may reduce the risk of AH via protection against development of insulin resistance and by virtue of its inverse association with obesity, while concurrently increasing risk of AH via its proliferative effects on breast tissue.

To our knowledge, no previous studies have investigated the association between circulating IGFBP-2 levels and risk of AH. A greater understanding of the development of AH might enhance our understanding of the origins of breast cancer and help identify women who would benefit from increased surveillance and screening. To this end, we investigated the association of IGFBP-2 with risk of incident AH in a large prospective cohort of postmenopausal women.

## 2. Materials and Methods

### 2.1. Study Population

This investigation was conducted as a case-control study nested within Women's Health Initiative-Clinical Trial (WHI-CT) [20]. Briefly, the WHI-CT recruited 68,133 postmenopausal women, aged 50–79, from 40 US clinical centers between 1993 and 1998. At baseline, subjects completed questionnaires regarding demographic and behavioral factors, medical history, and use of medications (including hormone therapy). Each woman also underwent a physical examination including measurement of height and weight and of waist and hip circumference and provided a fasting blood sample [21].

### 2.2. Histopathology

Every 6 months, participants in the trial completed medical questionnaires on clinical events including breast procedures. In the Benign Breast Disease Ancillary Study, which was conducted in all WHI clinical centers [22, 23], women who had undergone a breast procedure were asked to provide consent for retrieval of the resulting histological sections. The histological sections were reviewed by the study pathologist (DLP) who was blinded to randomization assignment in the clinical trials and to other exposure information. Benign lesions were classified using well-established criteria as nonproliferative lesions, proliferative lesions without atypia, or atypical (ductal and/or lobular) hyperplasia [24, 25]. In order to assess intrarater agreement, a repeatability study was carried out on 144 histologic sections which, following initial review, were assigned new identification numbers (to blind the pathologist to the results of the first set of readings) and then reviewed a second time. Assessment of agreement on histological classification as described above yielded a kappa of 0.6 (95% CI, 0.4 to 0.7), consistent with estimates found in other studies [26, 27].

### 2.3. Definition and Selection of Cases and Controls

Cases in our study were women diagnosed with incident BPBD with AH during follow-up in the WHI-CT. Of the 294 cases of AH ascertained, a total of 275 cases of BPBD with AH were included in the present study, consisting of all cases of atypical hyperplasia that had serum available. Controls were women who did not develop BPBD during the same follow-up period as that for the corresponding cases and who did not have an abnormal mammogram or abnormal clinical breast exam during the same period. Controls were selected from eligible participants using risk-set sampling (with replacement) [28] and were individually matched to their corresponding case on age at baseline (within 2 years), race (non-Hispanic white, black, Hispanic, Asian or Pacific Islander, other, or missing), randomization group (and intervention/nonintervention arm), and date of baseline blood draw (within one year). One control was selected for each of the cases, giving a total of 550 subjects.

### 2.4. Laboratory Methods

Laboratory testing was performed by the Pollak Assay Laboratory at McGill University in Montreal, Quebec, Canada. Serum IGFBP-2 levels were determined by enzyme-linked immunosorbent assay (ALPCO Diagnostics), which has an assay sensitivity of 0.2 ng/mL. All samples but one which did not have sufficient volume were retested as blind duplicates and showed strong correlations (Pearson *r* = 0.999).

### 2.5. Statistical Analysis

Differences in serum IGFBP-2 between cases and controls and between different subgroups were evaluated using one-way ANOVA. Correlations between IGFBP-2 and serologic data, age, and body mass index (BMI) were assessed using Spearman correlation coefficients among controls only.

Conditional logistic regression models were used to estimate odds ratios (OR) and 95% confidence intervals (CI) for the association of IGFBP-2 with risk of atypical hyperplasia. Analyses were first conducted with adjustment for age (years, continuous), and BMI (<25, 25–30, 30–35, ≥35 kg/m^2^). Adjusting for BMI as a continuous variable did not change the results substantially when compared to the four-category adjustment (data not shown). Additional adjustment for other variables (e.g., age at menarche, parity, OC use, energy intake, and physical activity) had no discernible impact on the effect estimates and these variables were not included in the final models. A second model was then run, additionally adjusting for serum levels of insulin, C-reactive protein, and adiponectin, as well as tertiles of estradiol among nonusers of HT only, with HT users as a separate category (as standard estradiol assays cannot accurately measure equine hormones present in most HT preparations). Analyses were also conducted after stratifying separately by hormone use at baseline (nonusers of HT, unopposed estrogen users, and estrogen plus progestin users), BMI based on the median in controls (<27.9 kg/m^2^, ≥27.9 kg/m^2^), and age based on the median in controls (<62, ≥62 years). These stratified analyses were conducted by introducing interaction terms into multivariate models that also included the main effect variables. We also reran analyses after restricting the study population to those without a history of diabetes (16 cases and 17 controls reported a history of diabetes). For all analyses, tests for trend were performed by assigning median values to each quartile and modeling these categories as a continuous variable. All hypothesis tests were two-sided and all analyses were done using the statistical software Stata S/E 13.0 for Windows (STATA Corporation, College Station, TX).

## 3. Results

Overall, baseline serum IGFBP-2 was higher in controls than in cases (Table 1). In cases and controls, IGFBP-2 was also significantly higher among those women with a BMI in the normal range (<25 kg/m^2^) than in those classified as overweight or obese (≥25 kg/m^2^) (Table 1). Furthermore, serum IGFBP-2 was higher in nonusers of HT at baseline and among those without a history of diabetes (Table 1). Serum IGFBP-2 level was inversely associated with BMI, waist circumference, and serum levels of insulin, CRP, and estradiol and was positively associated with age at baseline and serum level of adiponectin (Table 2).

Considering all subjects together, serum IGFBP-2 was associated with a decrease in risk of AH in the age- and BMI-adjusted model only, with those in the highest quartile of serum IGFBP-2 having a 49% decrease in risk compared to those in the lowest quartile (OR = 0.51; 95% CI = 0.29–0.90) (Table 3). This association was attenuated and was no longer statistically significant when models were further adjusted for insulin, adiponectin, C-reactive protein, and estradiol and when additionally restricted to those without a history of diabetes (Table 3). When analysing IGFBP-2 as a continuous variable (per 10 ng/mL increase), very similar results were observed, again with a statistically significant decrease seen with increasing serum IGFBP-2 (OR = 0.98; 95% CI = 0.97–1.00; *p* = 0.011) that was attenuated with adjustment for insulin, adiponectin, C-reactive protein, and estradiol (OR = 0.99; 95% CI = 0.97–1.01; *p* = 0.361), and when restricted to those without a history of diabetes (OR = 0.99; 95% CI = 0.97–1.01; *p* = 0.194).

When stratified by use of HT, there was a decrease in risk of AH with increasing serum IGFBP-2 that was evident only among nonusers of HT (Table 4), although a test for interaction was not statistically significant. In nonusers of HT, when considering the fully adjusted model and restricting the analysis to nondiabetics, women in the highest serum IGFBP-2 quartile had a 67% decrease in the risk of AH (OR = 0.33; 95% CI = 0.13–0.86) compared to women in the lowest quartile (Table 4). There was no association between IGFBP-2 and AH in users either of unopposed estrogen or of estrogen plus progestin.

The association of IGFBP-2 with BPBD also appeared to differ by BMI (Table 5), although again a test for interaction was not statistically significant. There was no association between IGFBP-2 and AH in women classified within the normal BMI range, whereas among women classified as overweight or obese serum IGFBP-2 was associated with a decreased risk of AH. Considering the fully adjusted model and restricting nondiabetics, obese or overweight women in the highest quartile of IGFBP-2 had a 57% decrease in risk of AH compared to obese or overweight women in the lowest quartile of serum IGFBP-2. A similar analysis stratified by median age (62 years) indicated that there were no appreciable differences in estimates when comparing those above 62 years to those below 62 years (data not shown).

## 4. Discussion 

To our knowledge, this is the first study to investigate the association between serum levels of IGFBP-2 and the risk of atypical hyperplasia of the breast. Overall, we found a nonsignificant decrease in risk of AH with increasing serum IGFBP-2. The inverse association was of borderline statistical significance in two, prespecified subgroups: nondiabetic, nonusers of HT and women who were overweight or obese.

Although no studies have previously examined the association between IGFBP-2 and risk of BPBD, there is some epidemiological evidence for a role of IGFBP-2 in breast cancer. An early study investigated the association between IGFBP-2 and postmenopausal breast cancer and found a reduction in risk with increasing serum IGFBP-2 [29], although further studies were not able to confirm these findings [30–33]. However, one of these studies did find evidence of a significant modification of the association of IGFBP-2 with risk of breast cancer by BMI, with those at or above 26 kg/m^2^ showing evidence of decreased risk of breast cancer with increasing IGFBP-2, an association not seen in women under 26 kg/m^2^ [33], whereas another suggested that there was a reduction in risk with increased IGFBP-2 when restricted to ER-positive breast cancer only [30]. In addition to these studies, a survival study amongst women with postmenopausal breast cancer found that relatively high serum IGFBP-2 levels were associated with increased survival and also found evidence of effect modification by BMI [34].

Findings from our study and from these previous studies in breast cancer are in contrast with previous experimental evidence suggesting that IGFBP-2 promotes cell proliferation and tumor growth [8]. This may be indicative of a dual role for IGFBP-2 in breast cancer development [10]. In some specific breast cancer cell lines IGFBP-2 has a proapoptotic effect [35, 36]. However, in other breast cancer cell lines IGFBP-2 acts as a survival factor, enhancing proliferative potential and protecting cells against chemotherapy-induced death [9]. Furthermore, upregulation of tissue IGFBP-2 has been associated with loss of PTEN, a frequent early event in breast tumorigenesis [7]. PTEN acts as a tumor suppressor gene, and loss of PTEN can cause activation of the Akt signaling pathway leading to increased cell proliferation and inhibition of apoptosis [7].

With respect to breast cancer and perhaps other obesity-related cancers such as colorectal cancer [37], the proliferative potential of IGFBP-2 may be countered by its role in obesity and adipocyte biology [38]. IGFBP-2 can inhibit adipogenesis in cells* in vitro*, both via inhibition of IGF-I [39] and also independently of IGF-1 by reducing preadipocyte proliferation and differentiation [19]. In addition, in animal models IGFBP-2 overexpression is protective against obesity and also protects against the development of insulin resistance and increases glucose sensitivity [39]. There is evidence for this same reduced susceptibility to obesity and improved metabolic profile in humans [40, 41]. Given that obesity is a strong risk factor for breast cancer and potentially also a risk factor for AH in postmenopausal women, it is plausible that IGFBP-2 could confer protection against breast cancer development via inhibition of the adipogenesis that leads to obesity. This is consistent with the inverse association of IGFBP-2 with serum estradiol levels [17]. Estrogen levels, which are increased in obese women, are thought to contribute to breast cancer development both through direct stimulation of breast tissue growth and through mutagenic metabolites of estrogen [42].

Our findings suggest that the effect of IGFBP-2 on risk of AH was modified by both HT use and BMI. The inverse association between serum IGFBP-2 levels and breast cancer risk observed previously seems to be restricted to women who were not using HT and to those women who are overweight or obese. Although suggestive, our study was underpowered to detect significant interactions between these subgroups with respect to risk of AH. A further limitation of this study was that IGFBP-2 was only measured once, at baseline. Repeated measurements over time might have resulted in more accurate estimates of serum levels. Also, the WHI-CT participants were a nonrandom sample of the population, which may limit the generalizability of our findings. However, because the results reported here demonstrate associations between a measured biological marker and risk of AH, it seems plausible that this marker may have similar effects in other populations. There is also the possibility of disease misclassification in this study, which may have biased our results towards the null. Major strengths of this prospective study were the collection of prediagnostic blood samples at baseline and ascertainment of subsequent (postbaseline) development of AH, thereby limiting bias.

In conclusion, the results of this study are suggestive of an inverse association between serum IGFBP-2 levels and risk of atypical hyperplasia of the breast. This reduction in risk was most evident among nondiabetic, nonusers of HT at baseline and among obese and overweight women, and the reduction in risk persisted after adjustment for associated serologic factors such as insulin and estradiol. However, these results should be interpreted with caution, as the lack of statistical significance could be due to the relatively small numbers of study subjects available for analysis or could indicate that no true association exists between IGFBP-2 and AH. Nevertheless, given the association between AH and breast cancer, if our finding that serum IGFBP-2 level is associated with reduced risk of AH is confirmed in further studies, this may suggest a role for IGFBP-2 in the early stages of breast carcinogenesis.

## Figures and Tables

**Table 1:** Serum levels of IGFBP-2 by case-control status and levels of other variables.

	*N*	Mean serum IGFBP-2 (IQR) in cases	*N*	Mean serum IGFBP-2 (IQR) in controls
*All subjects *	275	211.4 (147.9–315.7)	275	225.0 (149.7–347.9)
BMI				
Normal (<25 kg/m^2^)	90	315.5 (193.1–427.6)	79	320.5 (187.9–433.0)
Overweight/obese (≥25 kg/m^2^)	184	176.7 (135.3–257.4)	195	198.6 (137.4–297.3)
		*p*_difference_ < 0.001		*p*_difference_ < 0.001
HT Use				
Nonusers of HT	139	244.7 (147.1–341.0)	160	241.5 (174.8–376.4)
Unopposed estrogen	62	191.9 (143.7–315.4)	59	158.3 (127.0–311.4)
Progestin + estrogen	74	187.4 (148.8–262.9)	56	191.9 (140.9–296.7)
		*p*_difference_ = 0.04		*p*_difference_ = 0.004
History of diabetes				
No	259	219.6 (148.5–317.3)	258	229.8 (152.9–360.1)
Yes	16	185.0 (124.8–273.1)	17	157.7 (139.6–193.1)
		*p*_difference_ = 0.33		*p*_difference_ = 0.01
Age at baseline				
<Median (62 yrs)	140	189.5 (136.5–284.1)	137	187.9 (136.8–281.7)
≥Median (62 yrs)	135	246.3 (153.0–339.6)	138	277.9 (180.4–400.0)
		*p*_difference_ = 0.01		*p*_difference_ < 0.001
Race				
White	238	219.9 (149.5–328.5)	238	232.0 (154.7–362.8)
Black	22	145.6 (110.2–264.7)	22	181.1 (126.7–246.5)
Other	15	235.7 (153.0–283.5)	15	187.6 (145.8–347.9)
		*p*_difference_ = 0.08		*p*_difference_ = 0.05
Smoking status				
Never	134	214.8 (150.1–338.7)	149	235.8 (156.5–364.4)
Ever	141	211.4 (144.0–309.5)	126	205.0 (147.4–311.4)
		*p*_difference_ = 0.40		*p*_difference_ = 0.24

**Table 2:** Spearman correlations between IGFBP-2 and age, BMI, and levels of other serologic factors among controls^a^.

Factor	Age	BMI	Waist	Insulin	CRP	Adiponectin	Estradiol
IGFBP-2	0.25	−0.34	−0.37	−0.46	−0.37	0.49	−0.29

^a^All *p* < 0.001.

**Table 3:** Age- and multivariable-adjusted OR (95% CI) for associations between baseline level of IGFBP-2 and risk of atypical hyperplasia of the breast.

IGFBP-2 (*µ*IU/mL)	Co/Ca	Model 1 OR^a^	Model 2 OR^b^	Co/Ca	Model 3 OR^c^
Q1 (<149.9)	69/77	1.0^Ref.^	1.0^Ref.^	63/71	1.0^Ref.^
Q2 (150.0–225.4)	69/69	0.86 (0.53–1.39)	1.11 (0.65–1.87)	61/64	1.14 (0.65–1.98)
Q3 (225.5–349.5)	69/78	0.88 (0.55–1.40)	1.09 (0.65–1.84)	66/74	1.03 (0.60–1.78)
Q4 (≥349.6)	68/51	0.51 (0.29–0.90)	0.70 (0.36–1.38)	68/50	0.65 (0.32–1.31)
Trend		0.03	0.27		0.16

^a^Model 1 adjusted for age and BMI (<25, 25–<30, 30–<35, ≥35 kg/m^2^).

^b^Model 2 additionally adjusted for serologic level of insulin, adiponectin, C-reactive protein, and tertile of estradiol.

^c^Model 3 additionally adjusted for serologic level of insulin, adiponectin, C-reactive protein, and tertile of estradiol and restricted to those without a history of diabetes.

**Table 4:** Age- and multivariable-adjusted OR (95% CI) for associations between baseline level of IGFBP-2 and risk of atypical hyperplasia of the breast after stratification by hormone therapy (HT) use.

IGFBP-2 (*µ*IU/mL)	Co/Ca	Model 1 OR^a^	Model 2 OR^b^	Co/Ca	Model 3 OR^c^
Nonusers of HT					
Q1	28/38	1.0^Ref.^	1.0^Ref.^	25/35	1.0^Ref.^
Q2	42/25	0.44 (0.21–0.95)	0.47 (0.20–1.08)	38/22	0.48 (0.20–1.15)
Q3	43/45	0.65 (0.32–1.33)	0.72 (0.33–1.60)	40/41	0.66 (0.29–1.52)
Q4	47/31	0.40 (0.19–0.83)	0.38 (0.15–0.93)	47/30	0.33 (0.13–0.86)
Trend		0.06	0.11		0.06
Unopposed estrogen users					
Q1	24/20	1.0^Ref.^	1.0^Ref.^	22/19	1.0^Ref.^
Q2	11/19	2.16 (0.78–5.99)	2.51 (0.87–7.23)	8/17	2.42 (0.81–7.21)
Q3	12/12	1.11 (0.38–3.24)	1.27 (0.42–3.89)	12/12	1.12 (0.36–2.49)
Q4	12/11	0.67 (0.21–2.15)	1.54 (0.42–5.62)	12/11	1.37 (0.37–5.06)
Trend		0.28	0.79		0.94
Estrogen plus progestin users					
Q1	17/19	1.0^Ref.^	1.0^Ref.^	16/17	1.0^Ref.^
Q2	16/25	1.60 (0.64–3.98)	2.06 (0.79–5.38)	15/25	2.00 (0.76–5.25)
Q3	14/21	1.26 (0.45–3.52)	1.43 (0.48–4.28)	14/21	1.41 (0.47–4.24)
Q4	9/9	1.00 (0.30–3.31)	1.37 (0.38–5.00)	9/9	1.29 (0.35–4.75)
Trend		0.70	0.91		0.85

^a^Model 1 adjusted for age and BMI (<25, 25–<30, 30–<35, ≥35 kg/m^2^).

^b^Model 2 additionally adjusted for serologic level of insulin, adiponectin, C-reactive protein, and tertile of estradiol.

^c^Model 3 additionally adjusted for serologic level of insulin, adiponectin, C-reactive protein, and tertile of estradiol and restricted to those without a history of diabetes.

**Table 5:** Age- and multivariable-adjusted OR (95% CI) for associations between baseline level of IGFBP-2 and risk of atypical hyperplasia of the breast after stratification by body mass index (BMI).

IGFBP-2 (*µ*IU/mL)	Co/Ca	Model 1 OR^a^	Model 2 OR^b^	Co/Ca	Model 3 OR^c^
Normal BMI (<25 kg/m^2^)					
Q1	8/9	1.0^Ref.^	1.0^Ref.^	8/8	1.0^Ref.^
Q2	17/18	0.97 (0.29–3.18)	1.27 (0.36–4.51)	16/18	1.50 (0.40–5.61)
Q3	18/28	1.36 (0.42–4.41)	1.99 (0.55–7.26)	18/28	2.49 (0.65–7.22)
Q4	36/34	0.79 (0.25–2.46)	1.52 (0.42–5.52)	36/34	1.37 (0.37–4.99)
Trend		0.42	0.72		0.89
Overweight/obese (≥25 kg/m^2^)					
Q1	60/68	1.0^Ref.^	1.0^Ref.^	55/63	1.0^Ref.^
Q2	51/51	0.89 (0.52–1.52)	1.19 (0.67–2.14)	44/46	1.22 (0.67–2.25)
Q3	51/49	0.82 (0.48–1.37)	1.02 (0.57–1.82)	48/45	0.95 (0.53–1.73)
Q4	32/16	0.40 (0.19–0.84)	0.46 (0.19–1.08)	32/16	0.43 (0.18–1.04)
Trend		0.02	0.10		0.05

^a^Model 1 adjusted for age and BMI (<25, 25–<30, 30–<35, ≥35 kg/m^2^).

^b^Model 2 additionally adjusted for serologic level of insulin, adiponectin, C-reactive protein, and tertile of estradiol.

^c^Model 3 additionally adjusted for serologic level of insulin, adiponectin, C-reactive protein, and tertile of estradiol and restricted to those without a history of diabetes.

## References

[1] Silvera S. A. N., Rohan T. E. (2008). Benign proliferative epithelial disorders of the breast: a review of the epidemiologic evidence. *Breast Cancer Research and Treatment*.

[2] Worsham M. J., Abrams J., Raju U. (2007). Breast cancer incidence in a cohort of women with benign breast disease from a multiethnic, primary health care population. *Breast Journal*.

[3] Kabat G. C., Jones J. G., Olson N. (2010). A multi-center prospective cohort study of benign breast disease and risk of subsequent breast cancer. *Cancer Causes and Control*.

[4] Cote M. L., Ruterbusch J. J., Alosh B. (2012). Benign breast disease and the risk of subsequent breast cancer in African American women. *Cancer Prevention Research*.

[5] Lakhani S. R. (1999). The transition from hyperplasia to invasive carcinoma of the breast. *Journal of Pathology*.

[6] Rohan T., Kandel R., Franco E. L., Rohan T. E. (2002). Breast. *Cancer Precursors: Epidemiology, Detection, and Prevention*.

[7] Dean S. J. R., Perks C. M., Holly J. M. P. (2014). Loss of PTEN expression is associated with IGFBP2 expression, younger age, and late stage in triple-negative breast cancer. *American Journal of Clinical Pathology*.

[8] Baxter R. C. (2014). IGF binding proteins in cancer: mechanistic and clinical insights. *Nature Reviews Cancer*.

[9] Foulstone E. J., Zeng L., Perks C. M., Holly J. M. P. (2013). Insulin-like growth factor binding protein 2 (IGFBP-2) promotes growth and survival of breast epithelial cells: novel regulation of the estrogen receptor. *Endocrinology*.

[10] Hoeflich A., Reisinger R., Lahm H. (2001). Insulin-like growth factor-binding protein 2 in tumorigenesis: protector or promoter?. *Cancer Research*.

[11] Azar W. J., Zivkovic S., Werther G. A., Russo V. C. (2014). IGFBP-2 nuclear translocation is mediated by a functional NLS sequence and is essential for its pro-tumorigenic actions in cancer cells. *Oncogene*.

[12] Ko J. M., Park H. K., Yang S., Kim E. Y., Chung S. C., Hwang I. T. (2012). Association between insulin-like growth factor binding protein-2 levels and cardiovascular risk factors in Korean children. *Endocrine Journal*.

[13] Friedenreich C. M., Bryant H. E., Alexander F., Hugh J., Danyluk J., Page D. L. (2000). Risk factors for benign proliferative breast disease. *International Journal of Epidemiology*.

[14] Catsburg C., Gunter M. J., Chen C. (2014). Insulin, estrogen, inflammatory markers, and risk of benign proliferative breast disease. *Cancer Research*.

[15] Judd H. L., Shamonki I. M., Frumar A. M., Lagasse L. D. (1982). Origin of serum estradiol in postmenopausal women. *Obstetrics and Gynecology*.

[16] Key T. J. (2011). Endogenous oestrogens and breast cancer risk in premenopausal and postmenopausal women. *Steroids*.

[17] Juncker-Jensen A., Lykkesfeldt A. E., Worm J., Ralfkiær U., Espelund U., Jepsen J. S. (2006). Insulin-like growth factor binding protein 2 is a marker for antiestrogen resistant human breast cancer cell lines but is not a major growth regulator. *Growth Hormone and IGF Research*.

[18] Ruan W., Lai M. (2010). Insulin-like growth factor binding protein: a possible marker for the metabolic syndrome?. *Acta Diabetologica*.

[19] Rajpathak S. N., He M., Sun Q. (2012). Insulin-like growth factor axis and risk of type 2 diabetes in women. *Diabetes*.

[20] The Women’s Health Initiative Study Group (1998). Design of the Women's Health Initiative clinical trial and observational study. *Controlled Clinical Trials*.

[21] Hays J., Hunt J. R., Hubbell F. A. (2003). The Women's Health Initiative recruitment methods and results. *Annals of Epidemiology*.

[22] Rohan T. E., Negassa A., Chlebowski R. T. (2008). Conjugated equine estrogen and risk of benign proliferative breast disease: a randomized controlled trial. *Journal of the National Cancer Institute*.

[23] Cui Y., Page D. L., Chlebowski R. T. (2007). Alcohol and folate consumption and risk of benign proliferative epithelial disorders of the breast. *International Journal of Cancer*.

[24] Dupont W. D., Page D. L. (1985). Risk factors for breast cancer in women with proliferative breast disease. *The New England Journal of Medicine*.

[25] Schnitt S. J., Connolly J. L., Tavassoli F. A. (1992). Interobserver reproducibility in the diagnosis of ductal proliferative breast lesions using standardized criteria. *The American Journal of Surgical Pathology*.

[26] Sidawy M. K., Stoler M. H., Frable W. J. (1998). Interobserver variability in the classification of proliferative breast lesions by fine-needle aspiration: results of the Papanicolaou society of cytopathology study. *Diagnostic Cytopathology*.

[27] Bodian C. A., Perzin K. H., Lattes R., Hoffmann P. (1993). Reproducibility and validity of pathologic classifications of benign breast disease and implications for clinical applications. *Cancer*.

[28] Langholz B., Goldstein L. (1996). Risk set sampling in epidemiologic cohort studies. *Statistical Science*.

[29] Krajcik R. A., Borofsky N. D., Massardo S., Orentreich N. (2002). Insulin-like growth factor I (IGF-I), IGF-binding proteins, and breast cancer. *Cancer Epidemiology Biomarkers and Prevention*.

[30] Grønbæk H., Flyvbjerg A., Mellemkjær L. (2004). Serum insulin-like growth factors, insulin-like growth factor binding proteins, and breast cancer risk in postmenopausal women. *Cancer Epidemiology Biomarkers and Prevention*.

[31] Kaaks R., Lundin E., Manjer J. (2002). Prospective study of IGF-I, IGF-binding proteins, and breast cancer risk, in northern and southern Sweden. *Cancer Causes & Control*.

[32] Keinan-Boker L., de Mesquita H. B. B., Kaaks R. (2003). Circulating levels of insulin-like growth factor I, its binding proteins -1,-2, -3, C-peptide and risk of postmenopausal breast cancer. *International Journal of Cancer*.

[33] Muti P., Quattrin T., Grant B. J. B. (2002). Fasting glucose is a risk factor for breast cancer: a prospective study. *Cancer Epidemiology Biomarkers and Prevention*.

[34] Probst-Hensch N. M., Steiner J. H. B., Schraml P. (2010). IGFBP2 and IGFBP3 protein expressions in human breast cancer: association with hormonal factors and obesity. *Clinical Cancer Research*.

[35] Schütt B. S., Langkamp M., Rauschnabel U., Ranke M. B., Elmlinger M. W. (2004). Integrin-mediated action of insulin-like growth factor binding protein-2 in tumor cells. *Journal of Molecular Endocrinology*.

[36] Frommer K. W., Reichenmiller K., Schutt B. S. (2006). IGF-independent effects of IGFBP-2 on the human breast cancer cell line Hs578T. *Journal of Molecular Endocrinology*.

[37] Diehl D., Hessel E., Oesterle D. (2009). IGFBP-2 overexpression reduces the appearance of dysplastic aberrant crypt foci and inhibits growth of adenomas in chemically induced colorectal carcinogenesis. *International Journal of Cancer*.

[38] Boney C. M., Moats-Staats B. M., Stiles A. D., D'Ercole A. J. (1994). Expression of insulin-like growth factor-I (IGF-I) and IGF-binding proteins during adipogenesis. *Endocrinology*.

[39] Wheatcroft S. B., Kearney M. T., Shah A. M. (2007). IGF-binding protein-2 protects against the development of obesity and insulin resistance. *Diabetes*.

[40] Hu D., Pawlikowska L., Kanaya A. (2009). Serum insulin-like growth factor-1 binding proteins 1 and 2 and mortality in older adults: the health, aging, and body composition study. *Journal of the American Geriatrics Society*.

[41] Martin R. M., Holly J. M. P., Smith G. D., Gunnell D. (2006). Associations of adiposity from childhood into adulthood with insulin resistance and the insulin-like growth factor system: 65-year follow-up of the Boyd Orr cohort. *Journal of Clinical Endocrinology and Metabolism*.

[42] Yager J. D., Davidson N. E. (2006). Estrogen carcinogenesis in breast cancer. *The New England Journal of Medicine*.

